# Nivolumab-Induced Refractory Hemorrhagic Gastritis and Duodenitis Requiring Multimodal Biologic Therapy

**DOI:** 10.14309/crj.0000000000001782

**Published:** 2025-08-07

**Authors:** Roney Shibu, Kayla Scully, Waled Mohsen, Robert Mason, Sooraj Rajendran Pillai

**Affiliations:** 1 Royal Brisbane & Women's Hospital, Brisbane, Australia; 2 Pathology Queensland, Gold Coast University Hospital, Gold Coast, Australia; 3 Department of Digestive Diseases, Gold Coast University Hospital, Gold Coast, Australia; 4 Department of Medical Oncology, Gold Coast University Hospital, Gold Coast, Australia

**Keywords:** nivolumab, gastritis, duodenitis, infliximab, vedolizumab

## Abstract

Rising immune checkpoint inhibitor use in modern cancer therapy has increased the incidence of immune-related adverse events. Although isolated upper gastrointestinal immune-related adverse events including gastritis and duodenitis are uncommon, they may be severe and potentially life-threatening. The heterogeneous presentation of immune-related gastritis and duodenitis causes diagnostic and therapeutic challenges. We present a rare case of severe hemorrhagic gastritis and duodenitis occurring 2 months after completion of nivolumab therapy for stage III melanoma. The disease proved refractory to intravenous pantoprazole, steroids, and infliximab alone, ultimately requiring the addition of vedolizumab in the short term. Complete clinical and biochemical remission was achieved after 12 months of infliximab, which was ceased a month later. Following cessation of immunomodulatory therapy, there was no recurrence of disease at 12-month follow-up.

## INTRODUCTION

Immune-checkpoint inhibitors (ICI) have been a leading breakthrough in the management of both earlier stage and advanced cancers of many primary sites.^1^ ICI induce an antitumor immune response by attaching and inhibiting T-cell surface receptors to leverage regulatory systems of a patient's immune system to target and destroy tumor cells.^2^ ICI have a very broad side effect profile (immune-related adverse events [irAE]), arising from a relatively uninhibited immune system targeting patient's organs.^3^ irAE can affect any organ system including the gastrointestinal tract. Combination immunotherapies of anti-programmed cell death protein 1 (PD-1) antibodies and either anti-cytotoxic T-lymphocyte associated protein 4 or anti-lymphocyte activation gene 3 antibodies are associated with both higher rates and increased severity of irAE. Nivolumab is an IgG4 monoclonal antibody to the PD-1 receptor that prevents binding to its respective ligand, PD-L1.

Gastrointestinal irAE, including colitis, enteritis, esophagitis, and hepatitis, are seen in 7.7% of patients treated with nivolumab.^4^ Grading of severity of adverse events is based on clinical features rather than endoscopic or histological findings (Supplementary Table S1, http://links.lww.com/ACGCR/A39). However, endoscopic and histological scoring is useful to predict treatment course.^5^ Isolated upper gastrointestinal tract manifestations of gastritis and duodenitis are rare and are more often appreciated concurrently with enterocolitis.

Management of irAE often involves discontinuing immunotherapy temporarily or permanently, short-term corticosteroids and in severe or refractory cases, longer term corticosteroids, or additional immunomodulators.^6^ There is a paucity of evidence in the management of immune-related gastritis, given its rarity; however, there are growing data for the use of biologic therapies.^6^ This evidence is largely extrapolated from the management of immune-related colitis, where infliximab (antitumor necrosis factor [TNF]-alpha) or vedolizumab (α4β7 integrin inhibitor) are used for severe or refractory disease. We present a rare case of steroid refractory isolated hemorrhagic gastritis and duodenitis secondary to nivolumab, requiring multimodal biologic therapy with infliximab and vedolizumab.

## CASE REPORT

A 17-year-old adolescent boy had a recent diagnosis 16 months earlier of stage III cutaneous melanoma of the head and neck treated with wide local excision and regional lymph node clearance, followed by 12 months of adjuvant nivolumab (480 mg 4-weekly) completed approximately 2 months before presentation, with surveillance imaging at completion showing no evidence of local or distant recurrence. The patient was transferred from a local hospital to the nearest tertiary facility with new symptoms of frank hematemesis, presyncope (dizziness and blurred vision), and generalized upper abdominal pain. The patient had experienced subacute symptoms 6 weeks before admission with generalized abdominal pain, anorexia, and early satiety accompanied by 11 kg of weight loss over this period, progressive postprandial nausea, and nonbilious vomiting. There was no dysphagia, diarrhea, fecal urgency, fevers, or other infective symptoms. In addition, there were no recent medication changes or nonsteroidal anti-inflammatory drug or alcohol use. The patient's medical history was also significant for autism spectrum disorder and attention-deficit hyperactivity disorder, which led to additional challenges in clinical assessment, investigations, and treatment. There was no personal history to date of autoimmune disease. There was a maternal history of Crohn's disease.

On presentation, the patient was Glasgow Coma Scale 15, afebrile, and tachycardic at 130 beats per minute with a blood pressure of 120/83 mm Hg. All other vital signs were within normal limits. Physical examination was notable for generalized tenderness and mild distention of the abdomen without clear findings of peritonitis. Relevant laboratory findings included a hemoglobin of 156 g/L (reference range (RR) 135-175 g/L), white cell count of 10.9 ×10^9^/L (RR 4.0-11.0 ×10^9^/L), platelets of 307 ×10^9^/L (RR 150-400 ×10^9^/L), lactate of 1.3 mmol/L (RR 0.5-2.2 mmol/L), bicarbonate of 22 mmol/L (RR 20-33 mmol/L), C-reactive protein (CRP) of 18 mg/L (RR < 5.0 mg/L), albumin of 28 g/L (RR 32-47 g/L), and serum ketones of 1.3 mmol/L (RR < 0.6 mmol/L). Coeliac serology was negative, fecal calprotectin was 5800 μg/g (RR < 50 μg/g), and fecal microscopy and bacterial, viral, and protozoal polymerase chain reaction were negative. Abdominal computed tomography showed mild ascending colon wall thickening and prominent subcentimeter ileocolonic lymph nodes favoring either mild colitis or mesenteric adenitis. The stomach and small bowel were radiologically unremarkable. Although immune-related colitis was considered, the absence of diarrhea was unusual.

**Figure 1.**
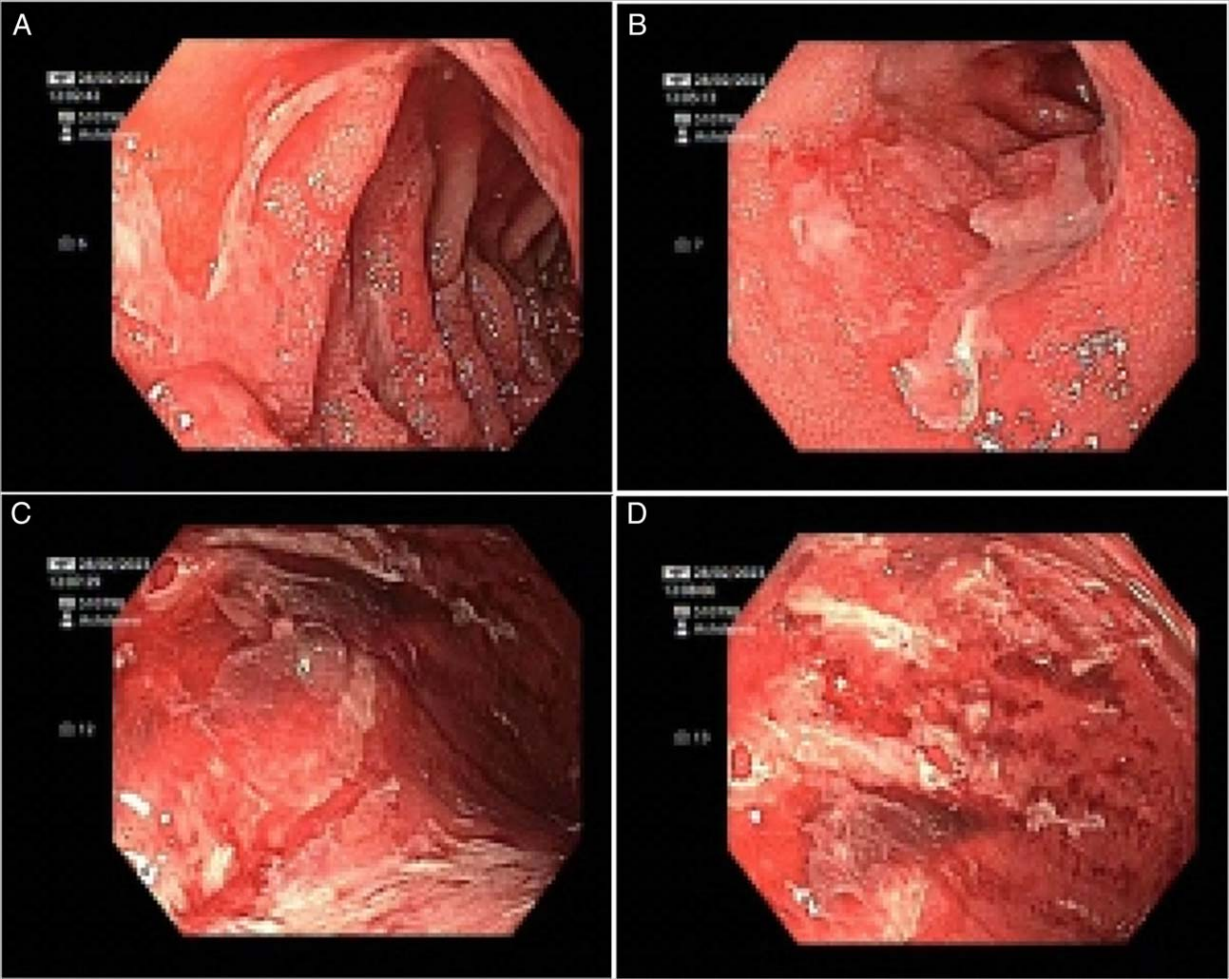
Severe inflammation characterized by erythema, congestion, erosions, and friability in duodenal bulb (A) and descending duodenum (B). Diffuse severe inflammation with hemorrhage characterized by erythema, congestion, mucositis, and mucus in the entire stomach (C and D).

Initial management included intravenous pantoprazole, antiemetics, and a free-fluid diet. Total parenteral nutrition was commenced due to concerns for severe malnutrition with starvation ketosis in setting of intolerance to oral and nasogastric nutrition and rising ketones (1.8 mmol/L). Urgent esophagogastroduodenoscopy (EGD) showed diffuse severe inflammation in the entire stomach and duodenal bulb with hemorrhage (Figure 1). The gastric biopsy showed ulceration, inflammatory exudate, and granulation tissue only. Duodenal biopsy showed erosion and severe active inflammation (active duodenitis). Immunohistochemistry for cytomegalovirus and *Helicobacter pylori* was negative. Flexible sigmoidoscopy was endoscopically and histologically normal. Colonoscopy was not performed at this time as the patient was intolerant of oral bowel preparation. Further laboratory evaluation was performed, including B12, anti-parietal cell antibodies, ANA, cANCA, and pANCA, to exclude autoimmune gastritis. These findings supported a diagnosis of severe immune-related hemorrhagic gastritis and duodenitis, a grade 4 irAE.

**Figure 2.**
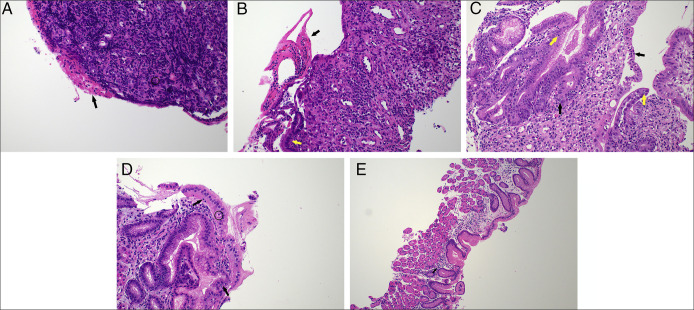
(A) Ulceration, inflammatory exudate (black arrow), granulation tissue (×200). (B) Active inflammation, ulceration (black arrow), and a focus of preserved gastric mucosa (yellow arrow) (×200). (C) Mixed pattern of inflammation showing both acute inflammation and intraepithelial lymphocytosis (×200). (D) Mild chronic inflammation. (E) Mild chronic inflammation (black arrow) (×100).

**Figure 3.**
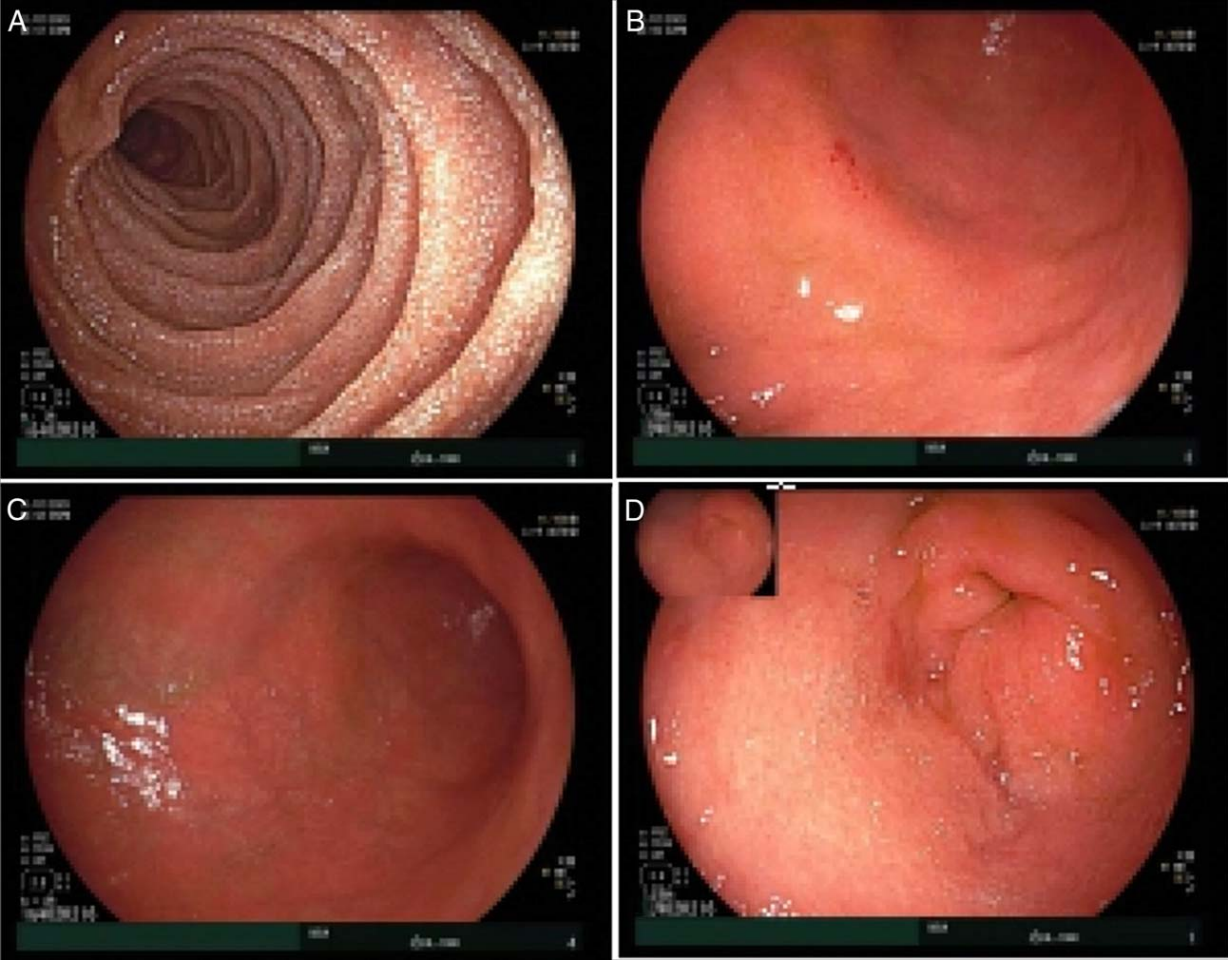
Normal endoscopic appearance of descending duodenum (A). Diffuse moderate inflammation characterized by erythema in entire stomach—gastric body (B-C) and gastric antrum (D).

**Figure 4.**
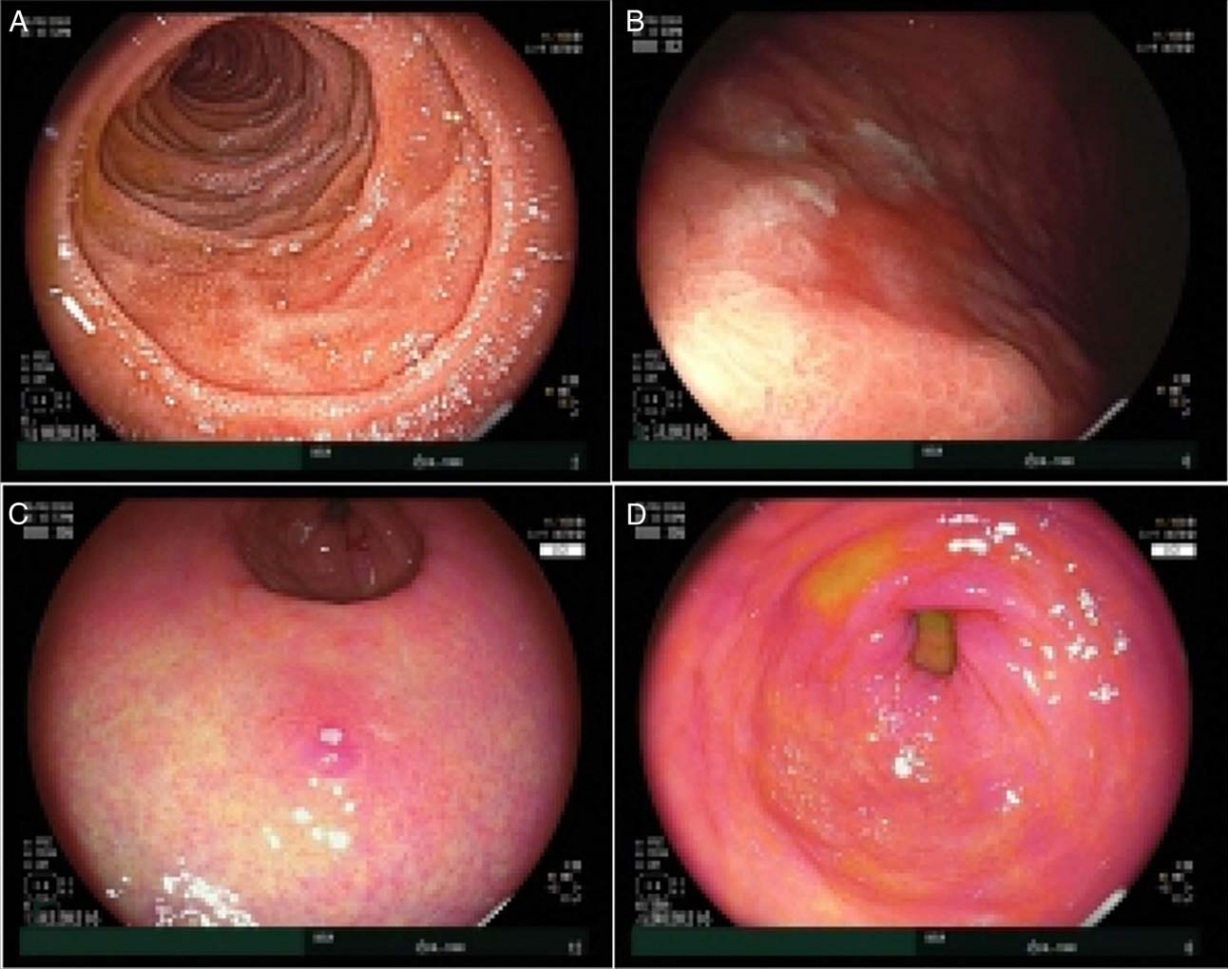
Normal endoscopic appearance of the descending duodenum (A). Significant improvement in gastritis with minimal inflammation in the gastric body (B), antrum (C), and prepyloric stomach (D).

Intravenous methylprednisolone (1 mg/kg) was commenced. After 72 hours of corticosteroid treatment, there was biochemical and clinical improvement with decrease in CRP to 6.9 mg/L and resolution of hematemesis; however, he remained intolerant of oral intake with ongoing abdominal pain and dry retching. Following 6 days of methylprednisolone, repeat fecal calprotectin increased to 8300 μg/g and CRP escalated to baseline (11.0 mg/L). Owing to lack of definitive clinical response, intravenous infliximab (5 mg/kg; total dose 400 mg) was commenced while continuing methylprednisolone. Ten days following the first infliximab dose, CRP and fecal calprotectin improved to 1.2 mg/L and 2000 μg/g, respectively. Repeat EGD 2.5 weeks after initial EGD revealed endoscopic resolution of duodenitis but persisting gastric inflammation. The gastric biopsy showed ulceration and granulation tissue (Figure 2A) while the duodenal biopsy showed normal histology, confirming the endoscopic assessment. Two further infliximab doses at 5 mg/kg were administered on day 11 and 16 after the initial dose, and a 10 mg/kg dose was given on Day 23. Trough infliximab level after 2 doses was >12 mg/L (RR 3.0-7.0 mg/L), and anti-infliximab antibodies were <10 ng/mL. Repeat EGD 5 weeks after initial EGD showed overall macroscopic improvement, but histology showed mucosal erosion with active inflammation in the gastric body biopsy and focal ulcer with active inflammation in gastric antral biopsy (Figure 2B). Despite CRP normalization, the albumin was 22 g/L, and there was persistent vomiting and lack of weight gain. As the patient demonstrated only a partial response following 4 doses of infliximab over a month, 300 mg of intravenous vedolizumab was administered. Within the next 5 days, there was resolution of vomiting and markedly improved oral intake and abdominal pain. However, clinical response to vedolizumab is usually slower, and therefore, the patient's response was likely largely driven by infliximab. Thus, 2 further 10 mg/kg infliximab doses were given on day 38 and 76, while vedolizumab was discontinued. At the time of discharge, CRP and fecal calprotectin were 2.0 mg/L and 1200 μg/g. He was discharged on a weaning course of prednisolone and 4-weekly 10 mg/kg intravenous infliximab. Macroscopic appearances on EGD at 2-month follow-up showed improvement (Figure 3), and histology showed mild increase in intraepithelial lymphocytes, mild active inflammation in the gastric body, and mild chronic gastritis in the antrum (Figures 2C and 2D). At 12-month follow-up, infliximab was ceased as there was clinical and biochemical remission with marked endoscopic (Figure 4) healing. Histology showed nonspecific mild chronic inflammation in the body (Figure 2E) and features of reactive gastropathy in the antral mucosa. Twelve months post cessation of infliximab, the patient remains in remission.

## DISCUSSION

Gastrointestinal irAE describe a spectrum of disease including upper and lower gastrointestinal manifestations. Lower gastrointestinal manifestations are more frequently reported, and isolated upper gastrointestinal manifestations of gastritis and duodenitis are rare.^7^ It is unclear why isolated upper or lower involvement occurs, although this heterogeneity may be partially explained by interplay of multiple factors. Key proposed drivers of gastrointestinal immune-related toxicity include antigen-specific T cells, impaired T-regulatory cell function, and inflammatory cytokines such as TNF-alpha.^8^ Immune-related colitis and gastritis are proposed to have shared etiopathogenic pathways. irAE more commonly involve barrier organs such as the gastrointestinal mucosa and skin, suggesting that impaired barrier tolerance leading to microbial and dietary antigen sensitization may drive toxicity.^9^ Gut microbiome dysbiosis is an ongoing area of research, but early evidence has linked certain microbiota profiles with higher risk of immune-related colitis,^10,11^ supporting this hypothesis. Personal or family history of autoimmune disease predisposes to developing irAE,^12^ and our patient's maternal history of Crohn's likely increased his genetic susceptibility. The presence of HLA-DQB1*03:01 allele has also been associated with the development of immune-related colitis,^13^ but HLA associations in immune-related gastritis have not been studied.

Immune-related gastritis is a diagnostic challenge due to the spectrum of clinical and endoscopic manifestations, and the absence of specific histological criterion. Endoscopic findings include mucosal erythema, edema, erosions, and friability, which are also seen in infection, medication-related gastritis, autoimmune gastritis, and Crohn's disease. The histological findings are nonspecific including diffuse chronic active gastritis, increased numbers of intraepithelial lymphocytes, and prominent apoptosis. Gastric periglandular inflammation (focally enhanced gastritis) and non-necrotizing granuloma have also been observed. Patients with severe symptoms can show ulcerative gastritis.^14–17^ The initial gastric biopsies showed florid active inflammation and ulceration, and no viral cytopathic changes or Helicobacter-like organisms were identified. The histological examination aided in excluding other specific etiologies. Together, these findings with the temporal association of symptom onset after nivolumab cessation and sustained remission off all immunomodulators supported a diagnosis of immune-related gastritis.

The clinical characteristics of upper gastrointestinal irAE are poorly understood. The time of onset of gastrointestinal irAE from ICI commencement varies significantly from 1 to 107.5 weeks with a median of 6 weeks.^6^ Onset of nivolumab-related gastritis is delayed compared with colitis, with a median time from treatment initiation of 6 months^18^ compared with 6-8 weeks.^19^ Our case manifested 14 months following nivolumab commencement and 2 months after nivolumab cessation.

Hemorrhagic gastritis is a severe and potentially life-threatening phenotype of immune-related gastritis, rarely described in literature. Our literature review revealed 4 cases of nivolumab-related hemorrhagic gastritis.^20–23^ All cases were receiving nivolumab at the time of symptom onset and demonstrated rapid resolution of symptoms with corticosteroids alone. A large pooled analysis of trials of ICI suggested gastrointestinal side effects generally present early in the course of anti-PD-1/PDL-1 therapy, yet the median onset of severe manifestations (> grade 3 irAE) was later.^24^ The pooled median time to resolution was only 3 weeks, following guideline-directed early initiation of immunomodulators in severe disease.^24^ Our case demonstrates that prolonged biologic therapy may be required for severe cases of immune-related hemorrhagic gastritis to achieve remission.

Given suspected similar pathophysiology and limited evidence, treatment of immune-related gastritis and duodenitis is extrapolated from the management of immune-related colitis.^6^ The recommended management for grade 4 immune-related colitis includes ICI discontinuation, intravenous corticosteroids, and consideration of biologic therapies for refractory disease. Infliximab is associated with rapid symptom improvement (often within 1-2 weeks), and complete symptom resolution may occur after a single infusion.^11^ The first randomized controlled trial evaluating infliximab for immune-related colitis demonstrated 90% clinical remission at 2 weeks after first infusion.^25^ Vedolizumab was commenced in our patient due to incomplete response to infliximab. Vedolizumab has lower response rates in patients with prior anti TNF-alpha exposure compared with those who are anti-TNF-alpha naïve.^26^ The rapid clinical improvement within days was also not typical for vedolizumab's mechanism of action, and thus, infliximab was likely the primary driver of response. Nonetheless, vedolizumab's alternative mechanism of action possibly partly contributed to achieving remission. Successful treatment of immune-related gastritis with vedolizumab has been reported only once.^27^ This is the first case of nivolumab-related gastritis requiring multimodal biologic therapy.^28^ Further research is required to define the spectrum of presentation and endoscopic findings, and optimal management strategies to achieve deep remission for immune-related gastritis/duodenitis. This will contribute to developing practice guidelines for management of upper gastrointestinal irAE.

## DISCLOSURES

Author contributions: W. Mohsen devised the project, identified key discussion points and R. Shibu drafted the manuscript. K. Scully and SR Pillai sourced and annotated the histology slides and contributed to discussion of histopathological diagnostic challenges. R. Mason contributed to the report from an oncology perspective. All authors critically revised the manuscript, approved the final version for submission and agree to be accountable for all aspects of the work. R. Shibu is guarantor of the article.

Financial disclosure: None to report.

Informed consent was obtained for this case report.

## Supplementary Material
