# Supplementary material for: Nivolumab-Induced Refractory Hemorrhagic Gastritis and Duodenitis Requiring Multimodal Biologic Therapy
Source: ACG Case Rep J. 2025 Aug 7;12(8):e01782. doi: 10.14309/crj.0000000000001782 (PMC12330355; doi:10.14309/crj.0000000000001782)
Supplement: Supplementary file 2 [file ac9-12-e01782-s002.docx]

| **Immune-mediated gastritis severity** | **Presentation** |
| --- | --- |
| Grade 1 irAE | Asymptomatic |
| Grade 2 irAE | Symptomatic; altered gastrointestinal function |
| Grade 3 irAE | Severely altered intake or gastric function; may require total parenteral nutrition |
| Grade 4 irAE | Life-threatening complications |
| Grade 5 irAE | Death |
